# Long Upper Pouch in Esophageal Atresia: A Rare Variant

**Published:** 2016-01-01

**Authors:** Enono Yhoshu, Jai Kumar Mahajan, Vedarth Dash

**Affiliations:** Postgraduate institute of Medical Education and Research, Chandigarh, India

**Keywords:** Esophageal atresia, Upper pouch, Esophageal stenosis

## Abstract

The earliest clinical sign of esophageal atresia (EA) is excessive salivation and the diagnosis is made by failure to pass an infant feeding tube (IFT) into the stomach. The diagnostic errors may occur due to presence of an unusually long upper pouch, when the IFT seems to pass into the stomach. We describe one such case and review the relevant literature.

## CASE REPORT

A 7-hour-old, full-term male neonate born to G1 mother by normal vaginal delivery was antenatally diagnosed to have an absent stomach shadow and associated polyhydramnios. The baby was presented to emergency with complaints of excessive salivation since birth. The insertion of infant feeding tube (IFT) showed coiling of the IFT at a distance of 18cm from the lower lip at the level of T-8 vertebra (Fig. 1). Contrast esophagogram showed dilated upper esophageal pouch seen ending just above the diaphragm (Fig. 2). However, the abdomen showed a normal gas pattern but the amount of gas was less than that of a usual patient of EA and TEF. A search was made for the associated anomalies. A 2D- echocardiography revealed small ASD (3mm) and PDA (5mm). With a provisional diagnosis of EA and TEF, the patient was taken up for rigid bronchoscopy. The bronchoscopy showed the presence of a fistula posteriorly at the level of carina (Fig. 3). The patient underwent thoracotomy through the 5th intercostal space in view of the caudally extending upper pouch and a lower location of the distal fistula. Both upper and lower pouches were overlapping each other for about 3cms. As the upper pouch was redundant, a part of it was resected in order to obtain a straight end-to-end anastomosis. Patient required postoperative ventilation for a period of 5 days. The anastomosis healed well and the patient was discharged on full oral feeds.

**Figure:**
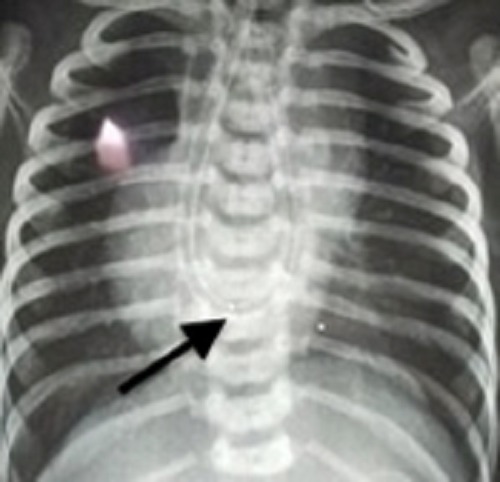
Figure 1: Infant feeding tube coiling at T8 (Arrow)

**Figure:**
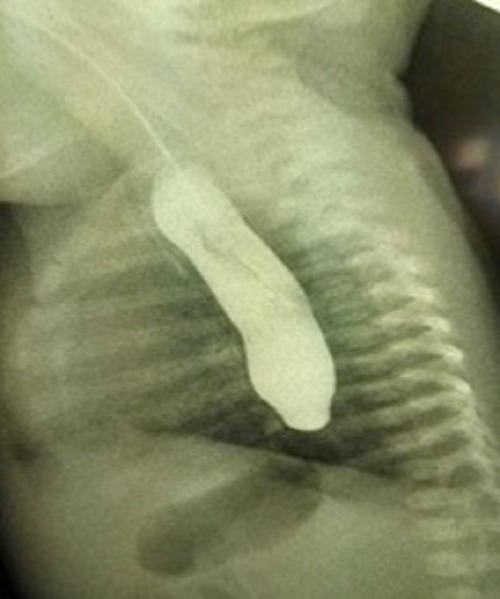
Figure 2: Contrast esophagogram showing dilated upper pouch ending at the level of diaphragm

**Figure:**
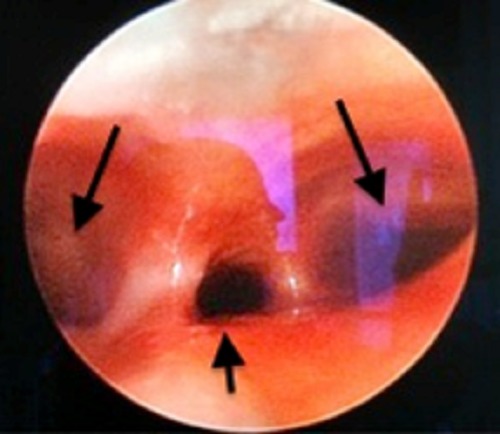
Figure 3: Bronchoscopy showing fistula opening at carina (small arrow) between left and right main bronchi (bigger arrows).

## DISCUSSION

Development of the esophagus seems to be more complex than just the cranio-caudal separation of the foregut into respiratory and esophageal components. Probably, the middle esophagus not only separates from the respiratory tract but also fuses with the proximal esophagus to complete formation of the esophageal tube.[1] What triggers the length of the upper pouch to vary from a short high pouch in the neck to a long pouch like the one seen in our case is unknown? Pediatric surgeons need to be aware of the variants of EA –TEF and the proceedings of the treatment. There are several case reports that describe about EA and TEF infants with a delay in diagnosis resulting from initial passage of an oral tube via the trachea through distal TEF into the stomach. [2,3] The authors stress on the importance using a stiff rubber catheter in place of a soft feeding tube, for diagnosis of EA. Similarly, the passage of infant feeding tube into long upper pouch gives an impression of it being in the stomach; however, the radiograph will show its position near the diaphragm. The contrast study delineates unusually long upper pouch. Preoperative bronchoscopy is particularly helpful to look for fistula, the presence of which is indicated by gas in the abdominal X-ray. Location of fistula may vary in such patients and prior endoscopic visualization is helpful during surgery. In addition, the bronchoscopy can also reveal abnormalities such as a laryngo-tracheo-esophageal cleft, tracheal stenosis, or other tracheal anomalies. [4-6]

## Footnotes

**Source of Support:** Nil

**Conflict of Interest:** Nil
